# Exploring Adverse Blood Donation Reactions Among Whole Blood Donors at a Tertiary Hospital Setting: A One‐Center Observational Mixed‐Methods Study

**DOI:** 10.1155/ah/3668746

**Published:** 2025-10-29

**Authors:** Redeemer Nana Fabea Addae, Patience Sobre, Inusah Abdul Mumin, Addo Samuel Siaw, Nichodemus Parker Quansah, Abigail Asmah-Brown, Prosper Junior Awittor, Joseph Boachie, Patrick Adu

**Affiliations:** ^1^ Department of Medical Laboratory Sciences, School of Allied Health Sciences, University of Cape Coast, Cape Coast, Ghana, ucc.edu.gh; ^2^ Blood Donation Collection Unit, Department of Medical Laboratory Services, Cape Coast Teaching Hospital, Cape Coast, Ghana, ccthghana.org; ^3^ Department of Medical Laboratory Science, Faculty of Health and Allied Sciences, KAAF University, Accra, Ghana

**Keywords:** adverse reactions, first-time donor, hematoma, vasovagal reactions, young donor

## Abstract

**Background:**

Although blood donation saves lives, it may be associated with adverse reactions. These reactions may be immediate or delayed and may be a factor in donor nonreturn. However, there is limited knowledge about blood donation–related adverse reactions and risk factors specific to the Ghanaian context.

**Aim:**

To determine the prevalence of adverse blood donation reactions and associated risk factors among successful blood donors at a tertiary hospital setting.

**Materials and Methods:**

This mixed‐methods research (May–June 2024) used observational techniques and semistructured questionnaires to investigate adverse reactions during and after blood donation. The study recruited 279 participants (241 mobile session donors and 38 in‐house donations) in a tertiary setting blood collection center. Bivariate logistic regression analysis was performed to evaluate the influence of some donor demographics on adverse reactions.

**Results:**

Overall, the prevalence of immediate adverse reaction was 44.4%; 51.4%, 36.5%, and 12.1% were local, vasovagal, and allergies, respectively. Blood donors who experienced adverse events had a statistically significantly lower median age (*p* = 0.007), lower systolic blood pressure (*p* = 0.004), lower diastolic pressure (*p* = 0.007), and lower weight (*p* = 0.030). Also, 22.1% of adverse donor reactions persisted 24 h postdonation; 38.7%, 58.1%, and 3.2% were local, vasovagal, and allergies, respectively. Bivariate logistics regression analysis showed that 16–19 years (aOR, 2.572, p = 0.477), males (aOR, 1.492, p = 0.125), students (aOR, 2.421, p = 0.325), the mobile session (aOR, 1.063, p = 0.928), and first‐time donors (aOR, 1.139, p = 0.690) were associated with nonstatistically significantly increased risk of immediate adverse donor reactions.

**Conclusion:**

There is an urgent need to build staff competencies and operationalize standard operating protocols to enable staff to identify and handle adverse blood donation events quickly. Further studies are needed to understand the factors responsible for the high prevalence of adverse donor reactions to inform improvements in blood donor care.

## 1. Introduction

Although donating blood is usually safe, local or systemic adverse events may occur during or after the process. Local adverse reactions are frequently associated with complications related to venous access, resulting in hematoma formation due to incorrect needle placement during venipuncture. However, systemic reactions can be classified as mild or severe, with vasovagal reactions being a common occurrence [1]. Studies have shown that the frequency of such events varies significantly, ranging from 0.6% to 36.0% [2, 3], and may occur immediately or postdonation [4, 5]. Such impact is especially noticeable among women, younger donors, and those donating for the first time [6].

Multiple research studies have indicated that individuals who experienced a negative event were less likely to revisit the blood donation center [7]. Therefore, it is crucial to avoid these adverse responses, especially in donors who are more prone to them [8]. As a result, the blood transfusion community has implemented some protective protocols to ensure the safety of blood donations and bolster public confidence in voluntary blood donation. Among these protocols, carefully selecting donors is particularly pivotal [9]. In addition, some health centers have adopted practices, such as staff training, predonation, and postdonation counseling, and established clear guidelines to increase donor retention to improve future contributions.

Despite the potential negative impact of adverse blood donation events on blood donor stocks, these investigations are insufficient to permit a comprehensive elucidation of the multiplicity of predisposition factors to enable tailoring of blood donor attention [10]. It is worth noting that developing countries still struggle to attract and retain blood donors or to ensure a sufficient and safe blood supply [11, 12]. The Ghana Blood Service has made continued efforts through awareness creation within communities and staff capacity‐building to boost donor retention and recruitment. However, data on the frequency of adverse donor events and factors associated with these adverse events in Ghana are scanty, presenting a knowledge gap in efforts to understand the perennial low blood stocks in Ghana. This mixed‐methods study used observational techniques and semistructured questionnaires to investigate adverse reactions during and after blood donation at a teaching hospital setting to provide empirical data to address this knowledge gap within the Ghanaian context.

## 2. Materials and Methods

### 2.1. Study Area/Study Population

The study was conducted at the Cape Coast Teaching Hospital (CCTH), Central Region, Ghana. CCTH serves as a referral center in the Central, Western, and Western North regions of Ghana. The blood collection center mobilizes blood through walk‐in donations and mobile blood mobilization drives. Overall, 279 successful individuals who donated whole blood were recruited into the study. The study was undertaken from May to June 2024; Ghana has two main seasons: rainy season (March–November) and dry season (December–February). Within the context of this study, blood donor refers to an individual who donated one unit (∼450 mL) of whole blood.

### 2.2. Study Design

This observational study employed a combination of observational techniques and semistructured questionnaires to investigate the adverse events experienced by successful donors following blood donation. The in‐house blood donation session took place at the CCTH blood collection center. Mobile donation sessions were conducted at Mfante Nyankomase Senior High School (SHS), Abakrampa SHS, Methodist SHS, and Amamoma S.D.A Church, all within the Central region of Ghana. The CCTH participants included both replacement and voluntary donors, while the mobile donation sessions primarily recruited voluntary donors. SHS students were the main focus of mobile blood mobilization sessions during the study period. Specifically, the observations of the blood donor bleeding processes were undertaken by trained research staff who utilized the Ghana Blood Services structured “Blood Donor Incident Report Form” [13] to systematically classify and document potential immediate blood donation adverse reactions among donors. After the blood donation, the blood donors were allowed to rest for 15–20 min; subsequently, a semistructured questionnaire was administered to capture detailed accounts of the donors’ subjective experiences and any adverse reactions they encountered postdonation. The 24 h post–blood donation adverse events were recorded through phone conversations and face‐to‐face interviews. However, whereas questionnaires were filled out by all 279 blood donors (documentation of all potential immediate adverse reactions), not all participants (particularly the SHS students) could be reached for the 24 h post–blood donation questionnaire.

### 2.3. Sample Size/Sampling Technique

This study used a nonprobability convenience sampling technique to consecutively recruit all blood donors donating whole blood at the CCTH (in‐house blood donation) or individuals who donated whole blood during the mobile blood mobilization drives organized by the CCTH Blood Mobilization Team. All the in‐house blood donations were mostly family replacement donations compared to the mobile sessions, which were all voluntary, nonremunerated blood donations. All participants were approached to volunteer for the study; however, only those who gave informed consent and had a successful blood donation were recruited into the study.

### 2.4. Inclusion/Exclusion Criteria

Successful donors, from 17 to 60 years of age, were recruited in this study. Blood donors who did not visit the CCTH and prospective donors who did not qualify for medical screening were excluded.

### 2.5. Ethical Consideration

This study is part of a broader study designed to characterize the blood donation process, such as donor screening, donor bleeding, post–blood donation care, blood donor retention, and the general blood donor health. The protocols have been approved by the Institutional Review Board of the University of Cape Coast (ethical clearance ID: UCCIRB/CHAS/2022/77), with the aspect regarding the experiences of blood donors with the existing blood donor collection within the Ghana health services previously published [12]. The research process upheld ethical standards, guaranteeing that participants were not coerced, and the evaluation posed no harm to their well‐being. In addition, consent forms and questionnaires for blood donors’ interviews were presented in simple language. The purpose, goal, risks, benefits, and funding of this study were explained to each participant before obtaining signed consent forms. Confidentiality was rigorously maintained through secure data management practices, preventing third‐party access to participant information. Although phone numbers were initially collected from participants who agreed to the 24 h post–blood donation follow‐up data collection, all such personal identifiers were removed after the follow‐up data collection. The report emphasized the broader implications for donor well‐being while respecting participants’ rights and ensuring the integrity of the research process.

### 2.6. Data Collection

#### 2.6.1. Observational Data Collection

Participants’ phlebotomies were closely monitored, and any signs or symptoms of potential adverse reactions were meticulously documented during and after the procedure. Furthermore, a semistructured questionnaire was administered to capture the donors’ personal experiences and any adverse reactions they may have encountered following the donation.

#### 2.6.2. Semistructured Questionnaire

The semistructured questionnaire (enclosed as Supporting file S1—Adverse donor reaction data collection instrument) employed in this study was the “Blood Donor Incident Report” form of Ghana’s National Blood Services (NBSG) guidelines for hemovigilance in Ghana [13]. The donor incident form captures the donor’s demographic data, donation information, donation‐related complications, and management/treatment of the adverse event sections. The donation‐related complication section is subdivided into a list of symptoms and signs related to vasovagal, local reactions, and allergies. This blood donor incident report form was used to document immediate adverse blood donation–related issues during (observations and recordings by the research team) and immediately after the blood donation (researcher assisted to curate subjective adverse events). Furthermore, blood donors were contacted over the phone 24 h post–blood donation to document answers related to the signs and symptoms itemized in the adverse blood donation section. For this study, any signs and symptoms recorded during (by researcher observations) and/or immediately after the blood donation (researcher‐assisted questionnaire filling) were classified as immediate; any adverse signs and symptoms recorded 24 h after the blood donation were classified as delayed.

### 2.7. Data Analysis

The data were analyzed using the computer Statistical Package for Social Sciences (SPSS Inc. 27.0) program. This program was a data analysis coding system that was employed to categorize information obtained from the questionnaire and personal observations. Categorical variables were compared using the Chi‐square test under a two‐tailed assumptions. In Table 1, the number of participants who experienced immediate and delayed adverse events is presented as per the demographic details of the participants. In Table 2, the characteristics of blood donors who did and did not experience immediate adverse donor events were compared using the Mann–Whitney *U*‐test. In Table 3, the specifics of the adverse events are presented as per the demographic details of the participants. As an individual blood donor may experience more than one adverse event, the numbers in Tables 1 and 3 are not necessarily the same. Furthermore, a bivariate logistic regression analysis was performed to identify the possible risks of adverse reactions. The 95% confidence intervals (CIs) and odds ratios (ORs) were calculated. In all statistical testing, a *p* value less than 0.05 denotes statistical significance under a two‐tailed assumption. In Supporting file S2, the number of times specific immediate and 24 h post–blood donation adverse events were recorded in blood donors is presented as percentages.

**Table 1:** The distribution of age^∗^ among the various demographics of whole blood donors in a mixed‐methods study undertaken from May to June 2024, in Cape Coast, Ghana.

	Total, *n* (%)	17–19 years *n* (%)	20–29 years *n* (%)	30–39 years *n* (%)	40–51 years *n* (%)
*Gender*					
Male	145 (52.5)	59 (40.9)	61 (42.1)	18 (12.4)	7 (4.6)
Female	131 (47.5)	89 (67.9)	41 (31.3)	1 (0.8)	0 (0.0)

*Occupation*					
Student	239 (86.3)	149 (62.3)	90 (37.7)	0 (0.0)	0 (0.0)
Others	38 (13.7)	0 (0.0)	12 (31.6)	19 (50.0)	7 (18.4)

*Venue*					
In‐house	43 (15.5)	0 (0.0)	19 (44.2)	17 (39.5)	7 (16.3)
Mobile session	234 (84.5)	149 (63.7)	83 (35.5)	2 (0.8)	0 (0.0)

*Donation history*					
First time	187 (67.5)	132 (70.6)	54 (28.9)	1 (0.5)	0 (0.0)
Repeat	90 (32.5)	17 (18.9)	48 (53.3)	18 (20.0)	7 (7.8)

*Note:* The data are presented as frequencies, n (%).

^∗^Two (2) participants did not state their respective ages, leading to *n* = 277 instead of 279.

**Table 2:** Blood donation–related adverse reactions experienced by whole blood donors in a mixed‐methods study undertaken from May to June 2024 in Cape Coast, Ghana.

	Immediate reactions				Donor reaction 24 h			
	Total, *n* (%)	Yes, *n* (%)	No, *n* (%)	*p* value	Total, *n* (%)	Yes, *n* (%)	No, *n* (%)	*p* value
^∗^Age (years)				**0.012**				0.154
17–19	149 (53.8)	75 (50.3)	74 (49.7)		22 (23.2)	8 (36.4)	14 (63.6)	
20–29	102 (36.8)	45 (44.1)	57 (55.9)		51 (53.7)	11 (21.6)	40 (78.4)	
30–39	19 (6.9)	3 (15.8)	16 (84.2)		15 (15.9)	2 (13.3)	13 (86.7)	
40–51	7 (2.5)	1 (14.3)	6 (85.7)		7 (7.4)	0 (0.0)	7 (100.0)	
Gender				0.635				0.849
Male	147 (52.9)	67 (45.6)	80 (54.4)		56 (58.9)	12 (21.4)	44 (78.6)	
Female	131 (47.1)	56 (42.7)	75 (57.3)		39 (41.1)	9 (23.1)	30 (76.9)	
Occupation				**< 0.001**				**0.042**
Students	241 (86.4)	117 (48.5)	124 (51.4)		64 (67.4)	18 (28.1)	46 (71.9)	
Others	38 (13.6)	7 (18.4)	31 (81.5)		31 (32.6)	3 (9.7)	28 (90.3)	
Venue				**0.002**				**0.034**
In‐house	43 (15.4)	10 (23.3)	33 (76.7)		37 (38.9)	4 (10.8)	33 (89.2)	
Mobile session	236 (84.6)	114 (48.3)	122 (51.7)		58 (61.1)	17 (29.3)	41 (70.7)	
Donation history				**0.015**				**0.037**
First time	188 (67.4)	93 (49.5)	95 (50.5)		40 (42.1)	13 (32.5)	27 (67.5)	
Repeat	91 (32.6)	31 (34.1)	60 (65.9)		55 (57.9)	8 (14.5)	47 (85.5)	

*Note:* The data are presented as the number of participants experiencing adverse events, *n* (%), as per the demographic characteristics of donor; statistical significance at *p* < 0.05 determined using the chi‐square test under two‐tailed assumptions; *p* values in bold indicate statistical significance at *p* = 0.05.

^∗^Two (2) participants did not state their respective ages (*n* = 277 for age category, instead of 279).

**Table 3:** Comparison of characteristics of blood donors who did and did not experience adverse events in a mixed‐methods study undertaken from May to June 2024, in Cape Coast, Ghana.

Variable	Age (years)	Systolic BP (mmHg)	Diastolic BP (mmHg)	Pulse	Hb (g/dL)	Weight (kg)
Adverse event	19	119	69.5	81	13.9	61
No adverse event	20	124	74	81	14.3	64
*p* value	**0.007**	**0.004**	**0.007**	0.572	0.100	**0.030**

*Note:* Statistical significance at *p* < 0.05 determined using the Mann–Whitney *U*‐test under two‐tailed assumptions; *p* values in bold indicate statistical significance at *p* = 0.05.

## 3. Results

The blood donor demographic data are presented in Table 1. The majority of the blood donors were in their teenage years (53.8%), or males (52.9%). Also, a higher proportion of the blood donors were students (86.4%) or recruited through a mobile session (84.6%). Furthermore, a major proportion (67.4%) of the donors were donating blood for the first time. A higher proportion of successful teenage blood donors were female (60.1%). Among the student population, teenagers also dominated, accounting for 62.3%. Additionally, during the mobile sessions, the majority of donors were teenagers compared to in‐house donations, representing 63.7%. Finally, most first‐time donors were teenagers, with a notable 70.6%.

The data were explored to determine the incidence of adverse reactions among successful blood donors (Table 2). Overall, whereas 86.0% (37/43) of in‐house blood donors could be reached for the post–blood donation data collection, only 24.6% (58/236) of the donors recruited from the mobile session (mostly high school students who have no access to mobile phones) could be reached for the post–donation data acquisition. After stratifying the data by age, there were significant differences (*p* = 0.012) in the proportion of donors experiencing immediate adverse reactions; adverse events decreased with increasing age of blood donors. Specifically, more donors aged 17–29 experienced adverse events compared to those aged 30–51. However, 24 h post–blood donation, the proportion of donors with persisting adverse reactions was statistically similar across age groups (*p* = 0.154). When the data were stratified by gender, there were no significant differences in adverse reactions between males and females immediately after donation (*p* = 0.635), or 24 h later (*p* = 0.849). Additionally, a significantly higher proportion of student donors experienced immediate (*p* ≤ 0.001) and 24 h post–donation adverse events (*p* = 0.042) compared to nonstudent donors. Most adverse events occurred during mobile blood donation sessions compared to in‐house sessions (*p* = 0.002). Immediate and delayed adverse reactions were notably higher among first‐time donors (*p* = 0.015 immediately, and *p* = 0.037 delayed).

The characteristics of the participants who experienced adverse events were compared to those who did not (Table 3). The blood donors who experienced adverse events had a statistically significantly lower median age (19 vs. 20 years; *p* = 0.007), lower systolic blood pressure (119 vs. 124; *p* = 0.004), lower diastolic pressure (69.5 vs. 74; *p* = 0.007), and lower weight (61 kg vs. 64 kg; *p* = 0.030). However, the median hemoglobin level or pulse rate did not differ significantly between the two groups.

Table 4 shows the specifics of adverse reactions recorded among participants in the study. Specifically, 51.4%, 36.5%, and 12.2% of the immediate adverse reactions were local injuries, vasovagal reactions, and allergies, respectively. However, vasovagal reactions accounted for 58.1% of persisting adverse reactions 24 h postdonation compared to 38.7% being local injuries. Among blood donors in the 17–29 years’ group, a higher proportion of adverse reactions that persisted after 24 h were vasovagal adverse reactions, although local adverse events were the major immediate reactions within this group. Also, in both males and females, although vasovagal reactions accounted for < 40% of adverse events, vasovagal adverse events accounted for > 50% in each group 24 h post–blood donation. Furthermore, although vasovagal reactions accounted for about 32.0% of immediate adverse reactions (40.0% in first‐time blood donors vs. 25.0% repeat donors), > 50.0% of the adverse event that persisted after 24 h were vasovagal reactions when data were explored as per the blood donation history. When the specificities of the immediate adverse donor reactions were explored (Supporting file S2), 66.3% of vasovagal reactions were due to blood donors feeling weak (23.4%), feeling warm (19.6%), dizziness (14.0%), and sweating (9.3%); however, 24 h post–blood donation, 77.3% of vasovagal adverse events that persisted comprised feeling weak (45.5%) and dizziness (31.8%). Also, immediate intense pain at the needle site (27.7%), weakness in the arm (21.3%), and warmth at the needle site (17.0%) comprised 66.0% of the immediate local injury adverse events; however, intense pain at the site of the needle comprised 80.0% of local injury adverse event that persisted 24 h post–blood donation (Supporting file S2). Furthermore, itching at the site of needle puncture (43.5%) and restlessness (39.1%) comprised 82.6% of allergy as immediate adverse event; however, one blood donor reported itching at the site of needle puncture 24 h post–blood donation.

**Table 4:** Stratification of adverse donor reactions into subtypes among whole blood donors in a mixed‐methods study undertaken from May to June 2024, in Cape Coast, Ghana.

	Immediate (*N* = 181)				24 h (*N* = 31)			
	Total *n* (%)	Vasovagal (*n* = 66)	Local injury (*n* = 93)	Allergies (*n* = 22)	Total *n* (%)	Vasovagal (*n* = 18)	Local injury (*n* = 12)	Allergies (*n* = 1)
^#^Age (years)								
17–19	110 (61.1)	45 (40.9%)	50 (45.5%)	15 (13.6%)	25 (80.6%)	14 (56.0%)	10 (20.0%)	1 (4.0%)
20–29	61 (33.9)	21 (34.4%)	33 (54.1%)	7 (11.5%)	6 (19.4%)	4 (66.6%)	2 (33.3%)	0 (0.0%)
30–39	6 (3.3)	0 (0.0%)	6 (100.0%)	0 (0.0%)	0 (0.0%)	0 (0.0%)	0 (0.0%)	0 (0.0%)
40–51	3 (1.7)	0 (0.0%)	3 (100.0%)	0 (0.0%)	0 (0.0%)	0 (0.0%)	0 (0.0%)	0 (0.0%)
Gender								
Male	100 (55.2)	35 (35.0%)	51 (51.0%)	14 (14.0%)	16 (51.6%)	9 (56.3%)	6 (37.5%)	1 (6.2%)
Female	81 (44.8%)	31 (38.3%)	42 (51.9%)	8 (9.8%)	15 (48.4%)	9 (60.0%)	6 (40.0%)	0 (0.0%)
Occupation								
Students	164 (90.6%)	65 (39.6%)	78 (47.6%)	21 (12.8%)	30 (96.8)	17 (56.7%)	12 (40.0%)	1 (3.3%)
Others	17 (9.4%)	1 (5.9%)	15 (88.2%)	1 (5.9%)	1 (3.2%)	1 (100.0%)	0 (0.0%)	0 (0.0%)
Venue								
In‐house	23 (12.7%)	3 (13.0%)	18 (78.3%)	2 (8.7%)	1 (3.2%)	1 (100.0%)	0 (0.0%)	0 (0.0%)
Mobile session	158 (87.3%)	63 (39.9%)	75 (47.5%)	20 (12.6%)	30 (96.8%)	17 (56.7%)	12 (40.0%)	1 (3.3%)
Donation history								
First time	133 (73.5%)	54 (40.6%)	61 (45.9%)	18 (13.5%)	20 (64.5)	12 (60.0%)	8 (40.0%)	0 (0.0%)
Repeat	48 (26.5%)	12 (25.0%)	32 (66.7%)	4 (8.3%)	11 (35.5)	6 (54.4%)	4 (36.4%)	1 (9.1%)

*Note:* The data are presented as frequencies of a particular adverse event subtype, *n* (%); note that, as some participants experienced more than one adverse event, the numbers in Table 2 are higher than the numbers in Table 1 (which report the number of participants experiencing adverse events).

^#^Indicates a missing data point as one participant did not state the age.

In Table 5, logistic regression was used to explore the association of donor demographics with the likelihood of immediate adverse reactions. The regression analyses showed that participants who were aged 17–19 years (aOR = 2.572, *p* = 0.477), males (aOR = 1.492, *p* = 0.128), students (aOR = 2.421, *p* = 0.325), recruited during mobile donation sessions (aOR = 1.063, *p* = 0.928), or first‐time donors (aOR = 1.139, *p* = 0.690) had marginally increased risk of immediate adverse donor reactions; however, none of these reached statistical significance.

**Table 5:** Possible risk factors of immediate adverse reactions among successful whole blood donors in a mixed‐methods study undertaken from May to June 2024, in Cape Coast, Ghana.

Variables	B	Wald	*p* value	aOR	95% C.I.
*Age (years)*					
17–19	0.945	0.505	0.477	2.572	0.190–34.816
20–29	0.804	0.395	0.530	2.234	0.182–27.449
30–39	0.122	0.010	0.922	1.130	0.097–13.159
40–51	Reference				

*Gender*					
Male	0.400	2.313	0.128	1.492	0.891–2.499
Female	Reference				

*Occupation*					
Students	0.884	0.968	0.325	2.421	0.416–14.104
Others	Reference				

*Venue*					
Mobile session	0.062	0.008	0.928	1.063	0.278–4.063
In‐house	Reference				

*Donation history*					
First time	0.130	0.159	0.690	1.139	0.601–2.156
Repeat	Reference				

*Note:* The data are presented as frequencies, *n* (%), with statistical significance at *p* < 0.05 determined using binary logistic regression analysis; B is the estimated coefficient/unstandardized regression weight of the regression model; Wald is the test statistic for the individual predictor variable derived from (B/S.E.)^2^.

Abbreviations: aOR, adjusted odds ratio; CI, confidence interval.

## 4. Discussion

Blood donation could potentially be associated with possible risks of adverse reactions [5], even though blood centers have largely underestimated such occurrences. Adverse events during blood donations are likely to reduce the chances of blood donor return and negatively impact donated blood stocks. Using a mixed‐methods approach, this study explored postdonation adverse reactions among successful blood donors at the blood collection center in a tertiary hospital setting to ascertain the frequency of immediate and delayed adverse reactions and potential donor‐specific risk factors. Our findings revealed that 44.4% of blood donors were susceptible to immediate adverse donor reactions with 22.1% persisting beyond the 24‐h mark. Relatedly, although vasovagal adverse events accounted for < 40.0% of immediate adverse events, vasovagal reactions accounted for > 50.0% of adverse events that persisted beyond 24 h post–blood donation. Also, blood donors who experienced adverse events had a statistically significantly lower median age, systolic blood pressure, diastolic pressure, and body weight. Logistic regression analysis demonstrated that teenagers, male gender, being a student, mobile blood donation, and first‐time donations increased the odds of adverse reactions experienced by donors. Overall, the evidence presented in this study suggests that adverse reactions are common among successful blood donors at the study center and should be addressed to enhance donor safety and encourage future donations.

Blood donation mobilization drives across high schools in Ghana provide an invaluable avenue to sustain blood stocks. It is interesting to note that although blood donors in Ghana are generally males, this study found that in blood donations organized in high schools, females comprised nearly half (47.1%) of the donors. This raises questions on what may be responsible for the male dominance in blood donation drives across the general population in Ghana as demonstrated in previous studies. While student‐centric donations are an invaluable resource in bloodstock sustenance, our adverse events recording also demonstrated that these adverse events occurred at a higher frequency in students and had an inverse association with blood donors’ age. Similar studies have also found a strong inverse association between age and adverse events [6, 14]. We identified the ignorance of the blood donation procedure and predonation screening results as a major source of fear, stress, and anxiety among young successful first‐time donors which triggered their adverse events [15]. As part of postdonation counseling, blood donors in this study were advised to have at least 6 h of sleep the night after the blood donation in line with a previous study that showed the benefit of adequate rest after blood donation [5]. Despite this, our 24‐h postdonation follow‐up indicated that some of the adverse events persisted beyond 24 h. It should be noted that although for this study, the authors intentionally followed up on blood donors to document potential adverse events, the blood collection center had no designated person whom blood donors could contact in the event of changes in body functions after the blood donation process. We believe this represents a potential leak in the ongoing efforts to improve blood donor retention, given that donors who might not have had the opportunity to discuss such changes in daily functions post–blood donation are less likely to return to donate. If blood collection centers make effort to retain some connections with the blood donors, through a dedicated healthcare professional helping blood donors address any postdonation concerns, this could foster trust building and improve donor retention. Contrarily, failure to provide expert care could lead to blood donors relying on misinformation that could disincentivize these donors from returning in subsequent times.

Prior studies have indicated a strong correlation between adverse reactions and gender [16], with a higher prevalence among female donors [6, 11]. We, however, found statistically comparable immediate and delayed adverse donor reactions in males and females. In addition, other studies found that young donors were vulnerable to syncope‐related falls and accidents, as well as vasovagal responses, after whole blood donation [17]. In this majority high school blood donor population, we found local injuries to be the most common immediate adverse reaction (46.7%, *p* < 0.001). However, the most persistent adverse reaction observed 24 h after donations were vasovagal episodes. Previously, Eder et al. reported that donors aged 16 and 17 had a 14‐fold higher risk of presyncope and vasovagal response–related injuries compared to donors aged ≥ 20 years [18]. Admittedly, any such vasovagal‐related events immediately after a blood donation exercise among these young blood donors may scar their willingness to engage in future blood donation exercises. There is, therefore, the need to build the capacity of blood donor recruitment staff to serve as contact persons able to provide medical counseling as part of postdonation care to improve postdonation experience. This will be an important step in retaining these young donors and consequently bolstering blood donations in Ghana and other sub‐Saharan Africa (SSA), given that blood donation mobilization drives across high schools have represented an invaluable source of bloodstock sustenance across SSA.

Several studies have consistently found younger donors, females, and first‐time donors as risk factors associated with higher adverse reactions among blood donors [19, 20]. It is important to note that differences in healthcare practices, donor screening protocols adopted in different study locations, and societal attitudes toward blood donation could influence the observed variation in the epidemiology of adverse donor events. This study recruited blood donors who had a multiplicity of the factors implicated in adverse blood donor reactions, such as first‐time donors, teenagers, and mobile session donors, and might have accounted for the nearly half experiencing adverse events. Admittedly, having adverse reactions can greatly reduce the chances of donating in the future [21] and should be avoided especially for more vulnerable blood donors [8]. Judging from our data, we believe this may be a more important area to address considering that vasovagal reactions (mostly feeling weak and dizziness), which has a higher propensity to deter future donor return [21, 22], persisted > 24 h post–blood donation. We believe that prioritizing staff training to appropriately handle donor adverse events during and after blood donation, donor education practices, and careful management of blood donors will likely improve blood donor experience and increase donor retention and future donations.

Despite highlighting a previously under‐recognized adverse blood donor reaction in the study area, our sampling frame was skewed toward a majority high school population and hindered interage group analyses. Also, the 24 h post–blood donation adverse event data collection was undertaken over the phone and therefore relied on participants’ self‐reporting and might not capture the full extent of adverse events. Moreover, there is always the possibility of bias in participants’ self‐reported data, which impacts the generalizability of the findings reported herein. Relatedly, as the majority of the mobile session donors were SHS students who largely do not use mobile phones at school, there was a high dropout rate in our 24‐h follow‐up data collection. Although we anticipate that future studies among non–high school blood donors may give more control over the study parameter estimates, we equally acknowledge that in SSA, where there is a high apathy to blood donation, such a sampling frame might not completely address the limitations highlighted herein. Furthermore, participants’ emotional/psychological state regarding experiencing adverse reactions was not accounted for in this study; only well‐screened healthy participants who had had predonation counseling were recruited in this study. Moreover, our data analyses were only performed in a bivariate way (e.g., reactions stratified by donor age or by occupation, but not for both at the same time); thus, the potential role of unequally distributed epidemiology/parameters could not be explored. Despite these acknowledged limitations, our study provides empirical data on high adverse blood donor reactions which may be a potential undercurrent negatively impacting blood donor retention strategies.

## 5. Conclusion

The high incidence of adverse blood donor reactions is indicative of a need to establish standard operating protocols to enable staff to quickly identify adverse events, and build staff competencies to handle these adverse events. Moreover, designating a resource person with effective communication competencies to follow up on successful donors will improve the blood donor experience and improve donor retention.

## Conflicts of Interest

The authors declare no conflicts of interest.

## Funding

This study did not receive any public funding; it was self‐financed by the co‐authors.

## Supporting Information

Additional supporting information can be found online in the Supporting Information section.

## Supporting information


**Supporting Information 1** Supporting file S1—Adverse donor reaction data collection instrument. The data collection instrument used to compile the adverse blood donor reaction in the research protocol titled “Exploring post‐donation reactions among successful blood donors in Cape Coast Teaching Hospital”. The data collection instrument was adopted from Ghana National Blood Services donor adverse reaction monitoring form.

**Supporting Information 2** Supporting file S2—Breakdown of the specificities of the various adverse blood donation events experienced by blood donors in a mixed‐methods study undertaken from May to June 2024, in Cape Coast, Ghana. This table documents (in percentages) the number of times specific immediate and 24 h’ postblood donation adverse events were recorded in blood donors. The adverse events were recorded under three main specificities: vasovagal reaction, local injury, and allergies. Each adverse event expressed by the blood donor was recorded as it is, even if two adverse events from the same specificity were experienced by a particular blood donor; no attempt was made by the researchers to subjectively use the dominant symptom to classify adverse reaction(s) under a single specificity.

## Data Availability

The data that support the findings of this study are available from the corresponding author upon reasonable request.
