# Supplementary material for: Exploring Adverse Blood Donation Reactions Among Whole Blood Donors at a Tertiary Hospital Setting: A One‐Center Observational Mixed‐Methods Study
Source: Adv Hematol. 2025 Oct 29;2025:3668746. doi: 10.1155/ah/3668746 (PMC12571990; doi:10.1155/ah/3668746)
Supplement: Supplementary file 1 — Supporting Information 1 Supporting file S1—Adverse donor reaction data collection instrument. The data collection instrument used to compile the adverse blood donor reaction in the research protocol titled “Exploring post‐donation reactions among successful blood donors in Cape Coast Teaching Hospital”. The data collection instrument was adopted from Ghana National Blood Services donor adverse reaction monitoring form. [file AH-2025-3668746-s002.docx]

**Adverse donor reaction data collection instrument (*adopted from Ghana National Blood Services donor adverse reaction montoring form^1^*)**

**Study title: *Exploring post-donation reactions among successful blood donors in Cape Coast Teaching Hospital***

| Donor Information | | | | | |
| --- | --- | --- | --- | --- | --- |
| Last Name: | | First Name (s) | | | 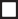 Male 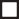 Female |
| Age: | Main Phone Number: | | Alternative Phone Number | Email address (optional) | |
| Occupation: | | | Location of Workshop/Residence: | | |

| Donation Information | | | | | |
| --- | --- | --- | --- | --- | --- |
| Date of Donation | Venue | | Donation History   - First Time - Repeat | Donation Number | Amount Bled (Ml) |
| Donor Category | Procedure Type | Voluntary Donation Panel Type | | | |

| Complications | | | | | | | |
| --- | --- | --- | --- | --- | --- | --- | --- |
| Date of Reaction | | Where did the complication/reaction happen? | | | | | |
| Time of Reaction | |  |  |  |  |  |  |
|  | **Related to Vasovagal Reaction**  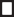 Cold extremities/Chills 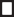 Sweating  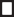 Feeling warm 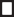 Dizziness  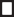 Loss of consciousness (<1min) 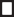 Loss of consciousness (>1min) 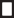 Nausea or vomiting  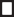 Twitching 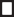 Convulsion  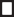 Loss of bowel/bladder control 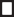 Feeling weak  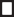 Hypotension mmHg 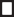 Slow pulse bpm  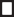 Rapid pulse bpm | | **Related to Local Injury**  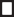Bright red blood  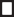 Haematoma Swelling  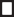 Immediate intense pain at the site 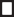 Warmth at site  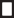 Numbness/tingling in arm 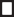 Shooting pain down arm 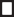 Weakness in arm  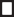 Pulse sensation in tubing  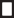 Rapid filling of bag (<4min) 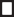 Red plasma  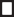 More than 1 needle prick  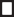 **Other sign or symptoms:** | | | | **Allergies**  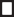 Restlessness  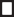 Generalized hives/rash/itching 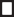 Itching at needle or bandage site 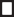 Rash/hives at needle or bandage site  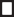 Redness at needle or bandage site  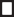 Shortness of breath Swollen throat/eyes/face  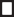 Wheezing 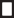 Chest pain |
| **Management** | | | | | | | |
| **Treatment** | | | **Vital Signs** | | | | **Outcome** |
| 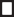 Pressure bandage applied 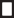 Cold compress applied 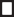 Warm compress applied  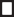 Made to take slow, deep breaths 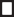 Made to breath into a paper bag 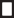 Feet elevated  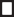 | | |  | **Time** | **BP** | **Pulse** | 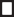 Recovered within 30 minutes 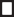 Recovered after 30 minutes 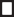 Partial recovery  **Release**  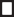Release, no escort 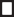Released, escort by |
|  |  |  | Pre- donation |  |  |  |  |
|  |  |  | Repeat 1 |  |  |  |  |
|  |  |  | Repeat 2 |  |  |  |  |

Further Information/Description

| Follow Up Record | | | |
| --- | --- | --- | --- |
| Date/Time | Type | By | Outcome and Remarks |
| After 24 hours | Phone ………………………………… Visit ……………………………………. |  |  |
| After 7 days | Phone ………………………………… Visit ……………………………………. |  |  |
|  | Phone ………………………………… Visit ……………………………………. |  |  |
|  | Phone …………………………………  Visit ……………………………………. |  |  |
|  | Phone …………………………………  Visit ……………………………………. |  |  |

| Diagnosis and Classification | | |
| --- | --- | --- |
| Localized  Haematoma Arterial Puncture Delayed Bleeding | Generalized  VVR without LOC VVR with LOC  VVR without injury  VVR with Injury:  VVR on collection site VVR off collection site    Severity  Mild (non-objective) Moderate  (objective) Severe (hospitalization) | Allergies  Local allergic reaction Generalized allergy/anaphylaxis |
| Tendon Injury  Nerve Irritation/Injury Duration: months  Painful Arm NOS |  | Other  …………………………………  ………………………………… |
|  |  |  |
| Localized inflammation  Other major vessel injury: |  | Imputability Definite Probable Unlikely Excluded |

Comments by Haemovigilance Lead

Reference

^1^NBSG-MoH-FDA, *Guidelines for haemovigilance in Ghana* 2022, National Blood Services Ghana, Ministry of Health and Food and Drugs Authority Accra, Ghana. <https://nbs.gov.gh/wp-files/Guidelines%20for%20Haemovigilance%20in%20Ghana_29-08-2022.pdf>
