# Supplementary material for: Exploring Adverse Blood Donation Reactions Among Whole Blood Donors at a Tertiary Hospital Setting: A One‐Center Observational Mixed‐Methods Study
Source: Adv Hematol. 2025 Oct 29;2025:3668746. doi: 10.1155/ah/3668746 (PMC12571990; doi:10.1155/ah/3668746)
Supplement: Supplementary file 2 — Supporting Information 2 Supporting file S2—Breakdown of the specificities of the various adverse blood donation events experienced by blood donors in a mixed‐methods study undertaken from May to June 2024, in Cape Coast, Ghana. This table documents (in percentages) the number of times specific immediate and 24 h’ postblood donation adverse events were recorded in blood donors. The adverse events were recorded under three main specificities: vasovagal reaction, local injury, and allergies. Each adverse event expressed by the blood donor was recorded as it is, even if two adverse events from the same specificity were experienced by a particular blood donor; no attempt was made by the researchers to subjectively use the dominant symptom to classify adverse reaction(s) under a single specificity. [file AH-2025-3668746-s001.docx]

**Supplementary table S2: Breakdown of the specificities of the various adverse blood donation events experienced by blood donors in a mixed-methods study undertaken from May to June 2024, in Cape Coast, Ghana.**

| **Immediate adverse events** | | | **24-hour adverse events** | | |
| --- | --- | --- | --- | --- | --- |
| **Vasovagal reaction** | **Local injury** | **Allergies** | **Vasovagal reaction** | **Local injury** | **Allergies** |
| Feeling weak (23.4%) | Immediate intense pain at the site (27.7%) | Itching at the needle site (43.5%) | Feeling weak (45.5%) | Intense pain at the site of the needle (80.0) | Itching at the needle site |
| Feeling warm (19.6%) | Weakness in the arm (21.3%) | Restlessness (39.1%) | Dizziness (31.8%) | Haematoma (13.3%) |  |
| Dizziness (14.0%) | Warmth at the site (17.0%) | Generalised itching; hives at the needle site; rash at the needle site (4.3%)* | Chills; dryness of mouth; feeling cold; severe headache; vomiting (4.5%)* | Swelling at the site of needle puncture (6.6%) |  |
| Sweating (9.3%) | Numbness, weakness in the arm (13.5%) |  |  |  |  |
| Twitching (8.4%) | >one needle prick; shooting pain in the arm (5.7%) |  |  |  |  |
| Chill (7.5%) | Tingling in the arm; rapid filling of bag (2.8%) |  |  |  |  |
| Rapid pulse (6.5%)* | Haematoma (2.1%) |  |  |  |  |
| Hypotension (2.8%) | Swelling; pulse sensation in the tube (0.7%) |  |  |  |  |
| Headache; loss of bowel control (1.9%)* |  |  |  |  |  |
| Loss of consciousness; cold at extremities; abdominal pains; loss of bladder control; convulsion (0.9%)* |  |  |  |  |  |

*indicates that the quoted percentage adverse event is not cumulative, but each named event occurred at the stated percentage; each adverse event expressed by the blood donor was recorded as it is even if two adverse events from the same specificity were experienced by a particular blood donor; no attempt was made by the researchers to subjectively use the dominant symptom to classify adverse reaction(s) under a single specificity.
